# Enhancing informed consent through multimedia tools in pediatric spinal surgery: a comprehensive review

**DOI:** 10.3389/fped.2024.1357462

**Published:** 2024-07-19

**Authors:** Marina Rosa Filezio, Nishan Sharma, Jennifer Thull-Freedman, Fabio Ferri-de-Barros, Maria J. Santana

**Affiliations:** ^1^ Department of Community Health Sciences, University of Calgary, Calgary, AB, Canada; ^2^ Department of Surgery, University of Calgary, Calgary, AB, Canada; ^3^ Department of Pediatrics, University of Calgary, Calgary, AB, Canada; ^4^ Department of Emergency Medicine, University of Calgary, Calgary, AB, Canada

**Keywords:** pediatrics, surgery, informed consent, multimedia tools, shared decision making, surgical discussion, patient-centered approach

## Abstract

Pediatric spine surgery is a high complexity procedure that can carry risks ranging from pain to neurological damage, and even death. This comprehensive mini review explores current best practice obtaining valid and meaningful informed consent (IC) prior to pediatric spinal surgery, including modalities that support effective comprehension and understanding. An evaluation of the literature was performed to explore understanding of surgical IC by patients or their guardians and the role of multimedia tools as a possible facilitator. The evidence discussed throughout this review, based on legal and ethical perspectives, reveals challenges faced by patients and guardians in achieving comprehension and understanding, especially when facing stressful medical situations. In this context, the introduction of multimedia tools emerges as a patient-centered strategy to help improve comprehension and decrease pre-operative uncertainty. This review highlights the need for a tailored approach in obtaining IC for pediatric patients and suggests a potential role of shared decision-making (SDM) in the surgical discussion process.

## 1 Introduction

Obtaining IC is a critical step prior to any surgical intervention and is both a medical necessity and a patient's legal right. It is usually obtained after a conversation between a physician and a patient (or guardian) that covers risks, benefits, alternatives, and a general overview of the proposed procedure (1).

To be considered valid, IC requires adequate understanding of the discussed content by the individual providing consent (2). Failure to provide valid consent not only compromises patient autonomy but also exposes them to possible hazards, potentially constituting medical malpractice or battery (3, 4). Use of multimedia tools during this process has been proposed as an effective strategy to improve understanding by patients and their guardians. This review focuses on fundamental components of a complete and accurate IC for pediatric patients undergoing spinal surgery.

## 2 Methods

A literature review was conducted to identify relevant studies using a search strategy that included the following keywords: “informed consent”, “surgical consent”, “multimedia tools”, “video-assisted consent”, “understanding”, “scoliosis”, “pediatric surgery”, “pediatric patients”, and “spine surgery”.

Databases searched included PubMed and Google Scholar, along with specific medical and legal databases such as the Canadian Medical Protective Association (CMPA) and the College of Physicians and Surgeons of Alberta (CPSA), and the Supreme Court of Canada published reports.

Study types included in the search were systematic reviews, prospective qualitative studies, randomized controlled trials, cross-sectional studies, and scoping reviews. These study designs were chosen to provide a comprehensive view of the topic, allowing for an assessment of both qualitative and quantitative aspects related to IC and the integration of multimedia tools to the IC process.

### 2.1 Inclusion criteria

1. Relevance to the topics of IC, multimedia tools, and their impact on patient and guardian understanding and satisfaction.
2. Publication in peer-reviewed journals or on reputable medical organization websites.
3. Written in English.
4. Availability of full text for comprehensive analysis.

### 2.2 Exclusion criteria

1. Studies not directly related to the topics of IC, multimedia tools, pediatric surgery, spine surgery, or patient and guardian comprehension.
2. Studies not published in English.

### 2.3 Search strategy

The search strategy involved a combination of relevant medical subject headings (MeSH) and the previously described keywords, tailored to each database. Boolean operators (AND, OR) were used to refine search queries. For example, in PubMed, the search string consisted of (“informed consent” OR “surgical consent”) AND (“multimedia” OR “video-assisted consent”). Study references were also reviewed to ensure complete search of the available literature. The search process took place in November 2023.

### 2.4 Study selection process and data extraction

The initial search provided 231 articles on PubMed and over 60,000 on Google Scholar. After removing duplicates, titles and abstracts of relevant articles were reviewed. Subsequently, full texts were obtained and assessed for eligibility based on the inclusion and exclusion criteria. Finally, the selected studies were subjected to data extraction and synthesis for the review.

Moreover, details such as study design, sample size, patient population (adult or pediatric), intervention methods, outcomes related to patient understanding and satisfaction, and key findings were abstracted and included in this review.

## 3 Results

### 3.1 Patient and guardian understanding of informed consent

#### 3.1.1 The informed consent process

IC is a mandatory prerequisite prior to medical procedure. It is performed to protect patients' legal rights and guide the ethical practices of medicine, safeguarding the patient's autonomy, self-determination, and inviolability (2, 5). This process includes a comprehensive dialogue between the physician and the patient or their guardian, covering four essential components: risks, benefits, alternative treatments, and general information about the proposed procedure (1, 6). Failing to obtain valid informed consent not only puts the patient's autonomy at risk but also poses possible danger to their safety, potentially constituting medical negligence or battery (3, 4, 6). To ensure compliance, a signed IC document serves as system-level proof to confirm that an appropriate consent process has taken place (5). By providing comprehensive information about potential risks, complications, and alternative treatments available, a valid IC process allows patients to make informed choices and take an active role in their healthcare (4). This is fundamental to making informed decisions that align with the patient's values and preferences, reducing the likelihood of misunderstandings or dissatisfaction.

The CMPA ascribes to the Oxford Dictionary definition of consent as a “voluntary agreement to or acquiescence in what another person proposes or desires; agreement to a course of action.” Deficient or inadequate surgical consent has at times been claimed by patients against physicians and can lead to allegations of assault and battery, when no consent was given at all, when the treatment provided was not the one discussed in the consent, or when the information provided by the physician to the consenter results in misrepresentation or lack of comprehension of the surgical procedure (6).

#### 3.1.2 The informed consent for pediatric patients

In pediatric settings, multiple steps must be considered to ensure that the consent obtained is valid and legal. The individual providing consent must be considered mature to provide consent (which is not always equivalent to the legal age of maturity of 18), and a determination of capacity as the ability to understand the information given regarding the proposed treatment, as well as its alternatives and possible consequences of a decision or lack of decision must be performed. In pediatric surgical setting, such capacity is either lacking, difficult to determine or varies with age, maturity, and specific situation (7–10). In cases involving complex procedures, such as pediatric spine surgeries, the parent or legal guardian of the patient often authorizes consent on behalf of the minor, acting in the best interest of the patient (6).

However, the matter of the patient's assent complementing the IC process should also be considered. The involvement of children and adolescents in medical decisions ought to align with their developmental capacity to comprehend the nature and implications of their medical condition, along with the risks and benefits associated with the proposed treatment and available alternatives. Physicians should respect expressions of assent or dissent, and recognize and assess instances of lack of capacity, minimizing potential harm while upholding the patient's best interests (9, 10).

For elective procedures, it is recommended to request valid IC in the clinic prior to the day of the surgery. This allows the patient and/or their guardian sufficient time to process the information received, reducing the likelihood of feeling pressure to proceed with a procedure that might not be fully understood (11).

#### 3.1.3 Understanding the informed consent prior to spinal surgery

Valid informed consent relies on the consenter's understanding of the provided information (1, 4). A prospective qualitative study on spinal surgery in adults highlighted key concerns expressed by patients regarding the IC process, including a desire for more detailed information on the procedure, its associated risks, benefits, and alternative treatments (12). Patients also demonstrated difficulty recalling potential risks and alternatives, suggesting that the availability of a video of the procedure might assist with their understanding (12).

Assessing a consenter's true understanding can be a complex task. Individuals may inaccurately report full comprehension due to factors like overestimating their understanding of the information, a desire to appear competent, or reluctance to admit confusion (13). Kreps (13) and Taherdoost (14) recommends the use of tests and questionnaires to directly and objectively determine patients' comprehension of the information provided. Validated questionnaires can assist with obtaining relevant, reliable, and valid information from patients and guardians submitted to IC processes (13, 14).

Schenker et al. presented a systematic review supporting the idea of including additional communication methods to improve comprehension of the written IC (3). This was supported by a systematic review by Glaser et al. in 2020, also mentioning the use of test/posttest components as a superior way of measuring consenter comprehension (1). Both publications emphasized the need for further studies in vulnerable populations.

#### 3.1.4 Understanding the surgical consent in the pediatric setting

In pediatric cases, generally an adult who holds guardianship of the pediatric patient is the one responsible for signing the consent, but often the patient is also involved in this process. Assessing both the guardian's and the pediatric patient's comprehension and satisfaction is crucial. The literature reveals that both tend to understand only a portion of the content presented, indicating a potential gap in the IC process.

Theologis et al. conducted a study evaluating the comprehension of pediatric spine patients and their guardians regarding the traditional verbal surgical consent process for Adolescent Idiopathic Scoliosis (15). Using a validated questionnaire, they measured understanding of the risks, benefits, expected outcomes, and overall comprehension of the process. The study revealed that both patients and guardians only understood approximately 60% of the content presented in the surgical consent, implying a potential gap in achieving a fully informed consent prior to pediatric spine surgeries.

### 3.2 The use of multimedia sources in supporting informed consent

#### 3.2.1 Traditional vs. multimedia

Traditional informed consent involves a discussion with a member of the medical team and a written consent form, and recent studies have focused on finding more patient-friendly approaches to IC delivery. Studies have suggested that additional information sources, beyond verbal or written communication, could enhance patients' understanding of surgical procedures, risks, and benefits (12, 16).

Video-assisted IC has emerged as a promising method to improve satisfaction and comprehension. A pilot study supported by The British Association of Spinal Surgeons found that the use of a video resource as a compliment to traditional preoperative consent (oral or written) significantly increased spinal patient satisfaction (17). Similarly, a study involving 142 patients showed that those who viewed an educational video as part of the IC process demonstrated higher knowledge scores and reported better comprehension and satisfaction compared to the control group receiving conventional consent (18).

#### 3.2.2 Multimedia tool as a patient-centered approach

IC plays a critical role in patient safety and care quality. Patient-centered approaches, like multimedia tools, have emerged as valuable tools to enhance IC processes and mitigate associated risks.

Abujarad et al. developed a patient-centered virtual multimedia IC tool, incorporating virtual coaching, multimedia library, and automated quizzes. Patient engagement was integral to its design and evaluation, resulting in a usable and meaningful tool (19, 20). Studies demonstrated the effectiveness and patient satisfaction with multimedia patient education tools and video consent, by improving comprehension and addressing patient concerns (17, 21).

#### 3.2.3 The use of multimedia tools in the pediatric setting

The use of multimedia tools in pediatric settings has shown promising results in enhancing comprehension and reducing anxiety in guardians during the IC process.

Tait et al. found that interactive multimedia programs significantly improved children's understanding of clinical trial information compared to traditional paper formats. Participants also reported finding the multimedia tool more effective and easier to follow (22). Additionally, a randomized controlled trial demonstrated that multimedia tools for pediatric surgical consent effectively reduced parental anxiety, leading to improved comprehension and overall satisfaction (23).

#### 3.2.4 The legal aspects of the surgical discussion and informed consent

The CMPA and CPSA provide a “standard of practice” to its members to ensure proper informed consent is obtained prior to medical procedures. It states that consent can be given in multiple ways, depending on the nature of the treatment or procedure. Written consent, in particular, is recommended for surgical and invasive procedures, and it should be documented in the patient's records (6, 24).

A report by Hanson & Pitt in 2017, based on a 5-year period, revealed that 65% of medical legal actions related to informed consent were related to surgery, and only 21% of these cases were decided in favor of the surgeon (25). The Supreme Court of Canada, in cases like Reibl v Hughes and Ciarlariello v Schacter, placed the responsibility of ensuring patient understanding on the physicians, confirming the duty outlined by professional organizations (26, 27). Physicians not only have to provide information about risks, benefits, and alternative treatments, but they also have a legal obligation to ensure patient comprehension (28).

Burningham et al. highlighted a significant gap between legal requirements and actual practice in surgical discussions and IC processes. Physicians are mandated to ensure full patient understanding, but both physicians and patients often struggle to recognize when comprehension is lacking. The study suggested that interventions, such as the use of multimedia resources, could enhance patients' knowledge of risks and help physicians fulfill their professional obligations (28).

#### 3.2.5 Patient-centered care and shared decision making

Adopting a patient-centered approach and SDM during the informed consent process improves collaboration and patient autonomy (2, 29). This approach considers evidence-based practices along with the patient's values and preferences. This process empowers patients to take an active and meaningful role in their healthcare decisions, along with their medical team (29, 30).

## 4 Discussion

The findings of this comprehensive review shed light on the critical aspects surrounding IC in surgical procedures, with a particular focus on the integration of multimedia tools, especially in pediatric settings such as pediatric spine surgery.

### 4.1 Enhancing understanding through multimedia tools

In the pediatric spine setting, where patients may have diverse cognitive abilities, the integration of multimedia resources in the IC process represents a pivotal advancement in patient-centered care. Studies consistently demonstrate that multimedia tools enhance patient comprehension of surgical procedures, associated risks, benefits, and alternative treatments (18, 19).

Multimedia resources provide a visual and auditory reinforcement of the information presented during the consent process, addressing various learning styles and cognitive abilities (1, 2, 31). Furthermore, these tools empower both patients and guardians, allowing them to engage actively in the decision-making process.

### 4.2 Implications for pediatric spine settings

In pediatric spine contexts, obtaining proper informed consent can be a complicated task. Even if a minor is deemed “mature” enough to fully understand the information given, the involvement of the guardian remains crucial due to their advocacy role (8, 10, 24). Stressful pediatric environments, like the PICU, can decrease comprehension and decision-making for parents and guardians (7). Tailored approaches, such as multimedia tools and pediatric SDM facilitators, appear to be valid approaches in enhancing IC.

A systematic review focusing on barriers and facilitators of pediatric SDM revealed that a frequently mentioned contributing factor is the utilization of well-crafted information specifically tailored for the pediatric setting. This includes the incorporation of lay language and integration of possible visual aids such as multimedia content (32).

Additionally, a randomized control trial found that multimedia tools were effective decreasing parental anxiety and improving comprehension of the IC process for guardians of pediatric patients being submitted to surgical procedures (23), such as pediatric spine surgeries.

### 4.3 Legal and ethical obligations

The legal and ethical obligations surrounding informed consent are clear. Physicians must ensure full comprehension of the proposed procedure, risks, and alternative treatments, and failure to meet this obligation is not only detrimental to patient experience but can also lead to legal consequences, including allegations of medical negligence or battery (3). Effective communication, improved by the use of multimedia tools, becomes crucial in bridging the gap between medical terminology and patient-friendly information, especially when involving pediatric patients in a SDM context.

### 4.4 Patient-centered care and shared decision making

The shift towards patient-centered care and SDM models aligns closely with the integration of multimedia tools in the IC process. This approach recognizes the importance of considering not only the medical perspective but also the patient's values, preferences, and individualized needs (2, 29, 30, 33). It may be useful to use shared decision-making to decide, together with the patient or their guardian, whether an invasive procedure is the right choice for their situation. If a patient or guardian decides that the procedure is likely to be the right choice, informed consent becomes a natural continuation of the decision-making process.

Studies have consistently demonstrated that patient engagement during the development of multimedia tools, illustrates a patient-centered approach that contributes to the creation of tools that are usable, acceptable, and meaningful to patients (19, 20).

### 4.5 Limitations and future directions

While the reviewed studies provide valuable insights, certain limitations should be acknowledged. Most published studies focus on adult populations, with fewer specifically tailored to pediatric spinal settings. This underscores the need for future research to address the gap in understanding the validity and applicability of multimedia tools to enhance IC process for pediatric spine patients and their guardians. Examining the validity of this method for both guardians and patients, features associated with effectiveness, and its potential role in facilitating pediatric assent are crucial areas to be further explored.

Finally, the long term-effects of multimedia-assisted IC remain an important area to be studied. Beyond initial comprehension and satisfaction, there is a need to investigate the impact of multimedia tools on post-operative satisfaction, outcomes, and patient-reported quality of life. By looking further into these elements, future research can provide more comprehensive understanding of the potential benefits of incorporating multimedia tools in the IC process in the pediatric spine surgical setting.

## 5 Conclusion

In conclusion, multimedia tools have been shown to be valuable in enhancing patient and guardian understanding and satisfaction during the IC process.

As healthcare continues to evolve, the thoughtful incorporation of multimedia resources represents an important step towards ensuring that pediatric spine patients and their guardians are active participants in the decision-making process.

Further research is necessary to evaluate the most effective and patient-preferred IC strategies and impact on immediate and long-term outcomes. Taking an evidence-based approach to improve informed consent demonstrates a commitment to patient-centered care and ethical medical practice.
